# 381. Transplantation of Maternal Intestinal Flora to the Newborn After Elective Cesarean Section - A Randomized Controlled Trial (SECFLOR)

**DOI:** 10.1093/ofid/ofae631.012

**Published:** 2025-01-29

**Authors:** Noora K Carpén, Willem M de Vos, Anne Salonen, Kaija-Leena Kolho, Sture Andersson, Otto Helve

**Affiliations:** HUS, Helsinki, Uusimaa, Finland; Human Microbiome Research Program, Faculty of Medicine, University of Helsinki, Helsinki, Uusimaa, Finland; Human Microbiome Research Program, Faculty of Medicine, University of Helsinki, Helsinki, Uusimaa, Finland; Pediatric Research Center, Helsinki University Hospital, University of Helsinki, Finland, Helsinki, Uusimaa, Finland; University of Helsinki, Helsinki, Uusimaa, Finland; The Finnish Institute for Health and Welfare, Espoo, Uusimaa, Finland

## Abstract

**Background:**

Normal microbial colonization process from the mother to the newborn is disrupted by birth by caesarean section (CS). We assess, whether in CS-delivered infants, the intestinal microbiome could be successfully and safely normalized by postnatal oral transfer of maternal fecal microbiome (FMT). In this double-blinded randomized controlled trial started in autumn 2019, we assess the difference in heterogeneity of the intestinal microbiome between the two groups from birth to 24 months of age.

The randomization code was opened in March 2024 when last transplanted child turned one year of age. Primary outcome measures are assessed from samples collected at 3 months of age.

**Methods:**

90 healthy pregnant women scheduled for elective CS were recruited. After screening and randomization, within two hours of delivery, 31 newborns were given 3.5 mg of the transplant from their own mothers or placebo mixed in mother’s milk.

The children are followed for 24 months. Stool and blood samples from the child and mother are collected during the follow up and analyzed for changes in microbiome heterogeneity between the groups and to assess, for example, immunological changes associated with the transfer.

**Results:**

All mothers were asymptomatic, no prescribed courses of antibiotics or travelling history outside the European Union region within three months of screening. 38 had positive findings in screening tests. 31 infants were included in the study of whom 15 received the transplant and 16 placebo. 3 adverse events were reported in the transplant group: one infant had thrombocytopenia at the age of two days, which normalized within one week. One infant was diagnosed with bilateral grade one intraventricular hemorrhage at the age of two weeks. One infant was treated for transient tachypnea of the newborn after receiving the transplant. None of the adverse events were considered related to the transplant.

**Conclusion:**

In our proof-of-concept study we previously demonstrated the effectiveness and safety of our protocol. The immunology and microbiota samples (fecal and blood) of our first-of-a-kind randomized controlled trial are being analyzed and first results will be available in September 2024.

**Disclosures:**

**All Authors**: No reported disclosures

